# Fruit Size Determines the Role of Three Scatter-Hoarding Rodents as Dispersers or Seed Predators of a Fleshy-Fruited Atacama Desert Shrub

**DOI:** 10.1371/journal.pone.0166824

**Published:** 2016-11-18

**Authors:** Claudia A. Luna, Andrea P. Loayza, Francisco A. Squeo

**Affiliations:** 1 Universidad de La Serena, Departamento de Biología, La Serena, Chile; 2 Instituto de Ecología y Biodiversidad (IEB), Santiago, Chile; 3 Centro de Estudios Avanzados en Zonas Áridas (CEAZA), La Serena, Chile; Hungarian Academy of Sciences, HUNGARY

## Abstract

Scatter-hoarding rodents can act as both predators and dispersers for many large-seeded plants because they cache seeds for future use, but occasionally forget them in sites with high survival and establishment probabilities. The most important fruit or seed trait influencing rodent foraging behavior is seed size; rodents prefer large seeds because they have higher nutritional content, but this preference can be counterbalanced by the higher costs of handling larger seeds. We designed a cafeteria experiment to assess whether fruit and seed size of *Myrcianthes coquimbensis*, an endangered desert shrub, influence the decision-making process during foraging by three species of scatter-hoarding rodents differing in body size: *Abrothrix olivaceus*, *Phyllotis darwini* and *Octodon degus*. We found that the size of fruits and seeds influenced foraging behavior in the three rodent species; the probability of a fruit being harvested and hoarded was higher for larger fruits than for smaller ones. Patterns of fruit size preference were not affected by rodent size; all species were able to hoard fruits within the entire range of sizes offered. Finally, fruit and seed size had no effect on the probability of seed predation, rodents typically ate only the fleshy pulp of the fruits offered and discarded whole, intact seeds. In conclusion, our results reveal that larger *M*. *coquimbensis* fruits have higher probabilities of being harvested, and ultimately of its seeds being hoarded and dispersed by scatter-hoarding rodents. As this plant has no other dispersers, rodents play an important role in its recruitment dynamics.

## Introduction

Seed dispersal and predation by scatter hoarding rodents are processes that influence seed survival probabilities, and hence are considered key for the establishment and maintenance of many plant populations [1,2,3,4]. Scatter-hoarders store fruits and/or seeds for later consumption in several caches located spatially apart [1,5]. In some cases, however, caches are forgotten and those seeds that are not recovered may have high survival and establishment probabilities [1,6,7]. Therefore, seed caching by rodents, which typically act as seed predators, can result in accidental seed dispersal, influence seedling recruitment probabilities and ultimately, plant distribution [2,4,5,8,9].

For seed dispersal by rodents to be advantageous to a plant, the benefits it gains by being dispersed must be higher than the losses incurred by the consumption of its seeds [10]. Consequently, the decisions rodents make while foraging can be particularly important [11]. In this sense, during the foraging process when rodents encounter a fruit or a seed, they must make several decisions: first whether to ignore or harvest the fruit or seed; second, whether to consume it *in situ* or remove it to another location and; third, if it is removed, whether to consume it immediately at the new location or store it for later consumption [12]. Several fruit and/or seed traits can influence these decisions [1] but seed size is generally recognized as one of the most important [9,10,13,14,15,16].

The relationship between seed size and rodent foraging behavior has been widely documented; typically, studies report that rodents harvest and hoard the largest seeds [10,15,17,18,19]. These preferences have been explained by the optimal foraging theory [20,21], because larger seeds offer higher nutrient content than smaller ones. In turn, manipulation and transport costs are also positively related to seed size and, depending on the size of the rodent, may impose upper limits to the seed sizes preferred [22,23] Therefore, to examine the role of fruit or seed size on the decisions rodents make while foraging, it is necessary to take rodent size into account, as it can influence the maximum fruit or seed size that can be removed [10,19,23,24].

Once at the hoarding or caching sites, fruit and/or seed size can also influence rodent behavior [17]. For example, large fruits, which presumably have higher pulp biomass than small fruits, may satiate rodents with their pulp before they consume their seeds [15,25,26,27], and thus increase the effectiveness of rodents as seed dispersers (“quick meal” hypothesis) [25]. Therefore, when fruits are large, there is a higher probability that rodents consume only the pulp—a reward rich in fat, protein carbohydrates and water [28,29]–and discard intact seeds [25,30]. Similarly, larger seeds may satiate rodents with only a fraction of their endosperm (i.e., satiation at the seed level) [31]; consequently, as seed size increases, there is a higher probability that the embryo remains intact, even if a seed has been partially predated [32]. This is particularly important for large-seeded species, as their seeds can often survive partial predation and contribute to plant regeneration [33–35].

*Myrcianthes coquimbensis* (Myrtaceae) is a shrub endemic to the Atacama Desert (Northern Chile) that produces large-seeded, fleshy berries that exhibit high size variation. Throughout its distribution range, fruits and seeds of *M*. *coquimbensis* are consumed and cached by three species of scatter-hoarding rodents differing in body size (from largest to smallest): *Octodon degus*, *Phyllotis darwini* and *Abrothrix olivaceus*. These rodents are the only vertebrates that consume and remove fruits and seeds of this plant; hence, they play a key role the recruitment dynamics of *M*. *coquimbensis* because their caches provide its only opportunity for seed dispersal [33].

In this study, we conducted cafeteria experiments to assess the role of fruit and seed size of *M*. *coquimbensis* on the decision-making process of rodents while foraging. Our goal was to determine whether: (1) fruit size influences the probability of a fruit being harvested and cached; (2) fruit choices are related to the size of the rodent and; (3) fruit and seed size influence the probability of seed predation. We predicted that rodents would harvest and hoard larger fruits because these provide higher nutritional rewards than smaller fruits. Second, because the three rodent species differ in body size, we predicted that the mean size of hoarded fruits would decrease with decreasing rodent body size and that the proportion of fruits hoarded would decrease as fruit size/rodent size ratio increased. Finally, we predicted that the probability of seed predation would decrease as a function of fruit size and that the percentage of seed mass loss due to consumption would decrease as a function of seed size.

## Materials and Methods

### Study species

*Myrcianthes coquimbensis* is an endangered shrub of the Myrtaceae distributed along an 83 km^2^ coastal strip in the southern limit of the Atacama Desert in Chile [36,37]. Its fruits are large-seeded berries, which depict wide variation in size both within and among individuals (mean ± SD: 3.52 g ± 2.67.g, range: 0.05–14.6 g, N = 1564) [38]. Fruits typically (i.e., 89% of the times) have only one large recalcitrant seed [38], but may occasionally contain up to four seeds. The number of seeds within each fruit can be visually determined without fruit destruction. In fruits that have only one seed, there is a strong relationship (r^2^ = 0.92, N = 456, *P* < 0.001) between fruit and seed mass [38], therefore the latter can be estimated relatively accurately from the former. The three rodent species that feed on *M*. *coquimbensis* are native to Chile and co-occur along its entire distribution range [33]. *A*. *olivaceus* (Cricetidae) is a small (30–33 g), diurnal, cave-digging omnivore that feeds on fruits, seeds and arthropods [39,40]. *P*. *darwini* (Cricetidae) is a nocturnal, omnivore-granivore that weighs approximately 60 g [38]. Finally, *O*. *degus* (Octodontidae) is an herbivorous caviomorph that builds underground burrows; it is primarily active during the early morning and late afternoon and weighs between 120 and 140 g [41–44]. Typically, these rodents forage for fruits underneath *M*. *coquimbensis*’ canopy; nonetheless, they may also climb on the plant and clip ripe fruits. When they encounter fruits on the ground, rodents can consume the pulp and seeds *in situ* or, alternatively remove fruits to hoarding sites (one at a time) [38]. Fruits are generally hoarded in rock cavities, where microclimatic conditions (i.e., low solar radiation, low soil temperatures and high soil humidity) promote the recruitment and early establishment of *M*. *coquimbensis* [45]. Although there are no studies that have examined the importance of *M*. *coquimbensis* on the diet of these rodents, it is likely an important food item, as well as a source of water, which is limited in this arid environment, because there are no other plant species that produce fleshy fruits in large quantities during the period fruits are available.

### Fruit collection

Between August and November 2014, we collected 900 fruits from at least 60 *M*. *coquimbensis* plants distributed in four populations. Fresh mass (g) was recorded for each fruit using an analytical balance (Scaltec SBC 31). Because there is a strong correlation (r^2^ = 0.92, n = 456, P <0.001) between fruit mass and fruit diameter [39], throughout this study we use mass as a measure of fruit size. Fruits collected included all size categories within the natural variation of this species (range 0.05–14.58 g). Because *M*. *coquimbensis* does not occur within the protected area system in Chile, and it is not included in the list of Chilean native woody plant species [46], no permits are required to collect its fruits.

### Rodent capture and maintenance

We captured 10 adult *A*. *olivaceus* (body mass, $\bar{x}$ ± se = 34.2 ± 2.0 g, range 24–43 g; 6 males and 4 females), 10 adult *P*. *darwini* (body mass, $\bar{x}$ ± se = 54.3 ± 5.5 g, range 30–76 g; 6 males and 4 females) and 10 adult *O*. *degus* (body mass, $\bar{x}$ ± se = 119.9 ± 10.9 g, range 84–180 g; 6 males and 4 females) with Sherman live-traps in two localities (Juan Soldado and Totoralillo) within *M*. *coquimbensis*’ distribution range. Given that learning can influence seed size preferences [23] and to ensure that rodents had foraging experience with *M*. *coquimbensis* fruits, trapping was conducted between August and November 2014, when the species was fruiting. We baited Sherman traps with oats, peanut butter and a piece of apple [47]. Fifty traps were set four times during the four-month period; on each occasion, we established them during the early morning and checked them every 12 hours for two consecutive days and nights. We carried captured rodents to Universidad of La Serena, where they were weighed. Following Muñoz and Bonal [23], prior to starting the experiments, rodents were acclimated by housing them in individual terrariums for 10 days, and providing them with hamster food, fruit and water *ad libitum*. All rodents were healthy during the experiments and when trials ended they were released at the point of capture. Capture and maintenance procedures were approved by the Ethics and Bioethics Committee of Universidad de La Serena (Informe de Ética/Bioética DIULS N° 048/13) and capture permits were obtained from the Servicio Agrícola y Ganadero de Chile (Chilean Agricultural and Ranching Service, Resolución N° 3723/2014).

### Fruit selection trials

**Fig 1:**
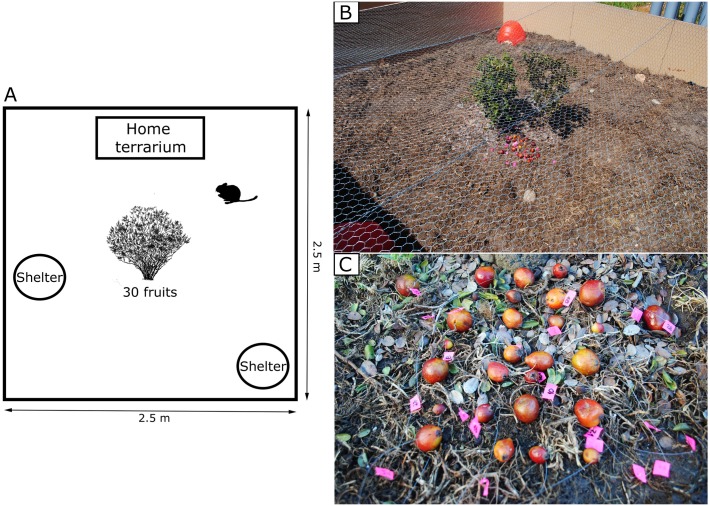
A. Schematic representation of the experimental arena. B. Arena showing the simulated *M*. *coquimbensis* shrub at its center with marked fruits underneath it; a shelter is also visible in one of the corners of the arena. C. *M*. *coquimbensis* fruits showing the fishing line and flagging tape.

We conducted trials outdoors in 2.5 x 2.5 x 0.5 m experimental arenas whose base was covered with 10 cm of soil. At the center of each arena, we placed freshly cut branches of *M*. *coquimbensis* (from individuals grown in greenhouses) to simulate a shrub, and at two sides of the arena we placed two plastic hamster houses to simulate sheltered sites and stimulate the rodent to hoard the fruits (Fig 1A) [23]. We performed 30 trials, with the 30 rodents captured; each trial lasted four days and rodents went through a trial individually and only once. Trials started once the home terrarium with the acclimated rodent was placed inside the experimental arena; for diurnal species (*A*. *olivaceus* and *O*. *degus*), trials begun in the morning (9:00 a.m.), whereas for *P*. *darwini* (the nocturnal species) trials started in the evening (6:00 p.m.). During each trial, we placed 30 *M*. *coquimbensis* fruits varying in size under the simulated shrub (Fig 1B). All fruits offered were previously weighed using an analytical balance and individually tagged with a 3 cm long (0.25 mm thick) fishing line, which had a small piece of flagging tape attached to one end (Fig 1C). The fishing line was inserted through the fruit (and seed) using a needle and tied at one end; hence, it remained attached to the seed even if the pulp had been eaten and the seed partially predated. Because the total weight added to the fruits was approximately 0.03 g, we did not expect the tag to alter patterns of fruit removal. The mean and range of fruit sizes offered to rodents was similar among species: *A*. *olivaceus* ($\bar{x}$ ± SD = 3.0 g ± 2.6; range: 0.05–12.1 g), *P*. *darwini* ($\bar{x}$ ± SD = 3.6 g ± 2.9; range: 0.12–13.9 g) and, *O*. *degus* ($\bar{x}$ ± SD = 3.3 g ± 3.2; range 0.08–14.6 g). At the end of the trial, we monitored the fate of each fruit and classified them as: ignored (fruits that were left intact at the encounter site) or harvested (fruits that rodents selected for consumption). In the latter case, fruits were categorized as (2) eaten at the encounter site (pulp and/or seeds of these fruits were consumed under the simulated *M*. *coquimbensis* shrub) or (3) hoarded (i.e., fruits and seeds stored in different locations within the experimental arena and hence considered dispersed). Finally, for all fruits that had been hoarded, we recorded whether they were intact, if the fleshy pulp had been eaten or if the seed had also been predated, in which case we calculated the percentage of seed mass loss. For this calculation, we estimated initial seed size using the parameters from the linear regression between fruit and seed mass.

### Statistical analyses

To examine whether the probability of a fruit being (1) harvested and (2) hoarded depended on its size and differed among rodent species, we used mixed effects logistic regressions and tested for significance using likelihood-ratio tests. For these analyses, the binary response variables were (1) if a fruit was harvested (yes/no) and (2) if a fruit was hoarded (yes/no), respectively. The independent variables were fruit size and rodent species, and the random factor was each individual rodent. We conducted the first analysis considering the entire set of fruits offered in each trial (N = 30), but for the second analysis we used only the subset of fruits that had been harvested, given that a rodent will decide which fruits to hoard only from those it has harvested [48]. We tested whether larger fruits would satiate rodents with only their pulp with the same model structure. In this case, the explanatory variables were fruit size and rodent species, and the response variable was if either discarded or hoarded seeds had some level of predation (yes/no). To model the seed size kernel (i.e., the probability distribution of a seed of any given size being dispersed) based on fruit size and examine whether each rodent species could transport the entire range of fruit sizes, we used the “mykernel” function in R (available at https://github.com/pedroj/dispkernels) considering all fruits that had been transported. We determined if the size of hoarded fruits differed among rodents using a mixed model, with fruit mass and rodent species as the dependent variable and independent variables, respectively and each individual rodent as the random factor. To examine whether seed choices of *A*. *olivaceus* and *P*. *darwini* are influenced by the ratio of fruit size to rodent size we first categorized all fruits in each individual trial into one of 10 fruit size/rodent size ratio categories, which ranged from 0.05 to 0.5. We then calculated the total percentage of fruits offered per ratio category for each rodent species. Afterwards, we performed an ANCOVA with the percentage of fruits removed in each ratio category as dependent variable (standardized by the availability of fruits per category), the rodent species as the independent variable and the fruit size/rodent size ratio as the covariate. We excluded *O*. *degus* from this analysis because, given its size, the largest *M*. *coquimbensis* fruits accounted for only 15% of its body mass. Finally, to determine whether the percentage of seed mass loss due to consumption decreases as a function of seed size, we performed a linear mixed model in which the dependent variable was the percentage of seed mass loss, the independent variables were initial seed mass and rodent species, and the random variable was each individual rodent. All statistical analyses were performed using the R statistical environment [49].

## Results

### Does fruit size influence the probability of being harvested and hoarded?

**Fig 2:**
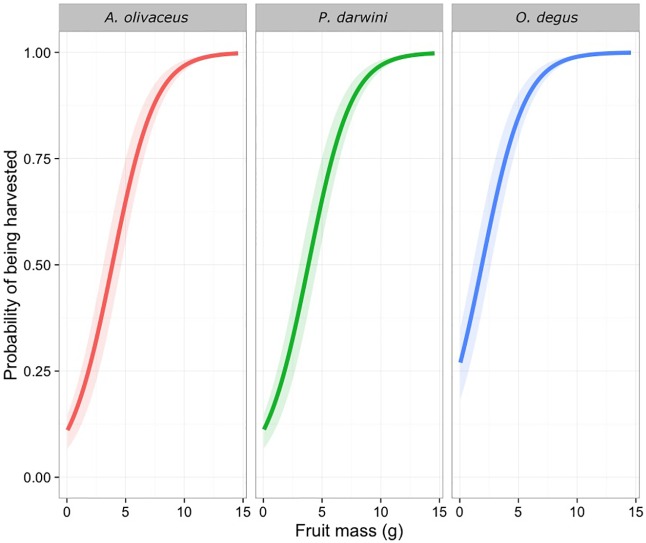
Relationship between fruit mass and the probability of a fruit being harvested for consumption by *A*. *olivaceus*, *P*. *darwini* and *O*. *degus*. The results were modeled using a mixed effects logistic regression.

We found that size affected the probability of a fruit being harvested (χ^2^ = 330.8, d.f. = 1, *P* < 0.001). Specifically, the probability of being harvested increased with fruit size for all rodent species (Fig 2). Sizes of selected fruits differed among rodents (χ^2^ = 39, d.f. = 2, *P* < 0.001) and among individuals (AIC = 895); for *A*. *olivaceus* and *P*. *darwini* (the smallest species), fruits had a 50% chance of being harvested if they weighed at least 3.5 g. In contrast, for *O*. *degu*, which is four- and two-fold larger that *A*. *olivaceus* and *P*. *darwini*, respectively, a fruit had a 50% or higher probability of being harvested fruits if it weighed at least 1.8 g.

**Fig 3:**
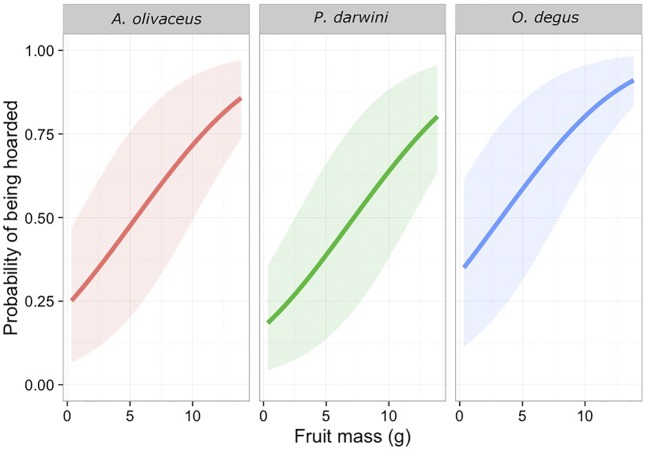
Relationship between fruit mass and the probability of a fruit that was harvested for consumption to be transported to a hoarding site by *A*. *olivaceus*, *P*. *darwini* and *O*. *degus*. The results were modeled using a mixed effects logistic regression.

Size also affected the probability of a fruit being hoarded (χ^2^ = 36.5, d.f. = 1, *P* < 0.001); larger fruits had higher probabilities of being hoarded than smaller ones (Fig 3). The size of fruits hoarded differed among rodents (χ^2^ = 13.8, d.f. = 2, *P* = 0.001) and among individuals (AIC = 612). Fruits had a 50% or higher probability of being hoarded by *A*. *olivaceus*, *P*. *darwini* and *O*. *degus* if their mass was at least 5.3 g, 6.5 g and 3.5 g, respectively.

### Are fruit choices related to the size of the rodent?

**Fig 4:**
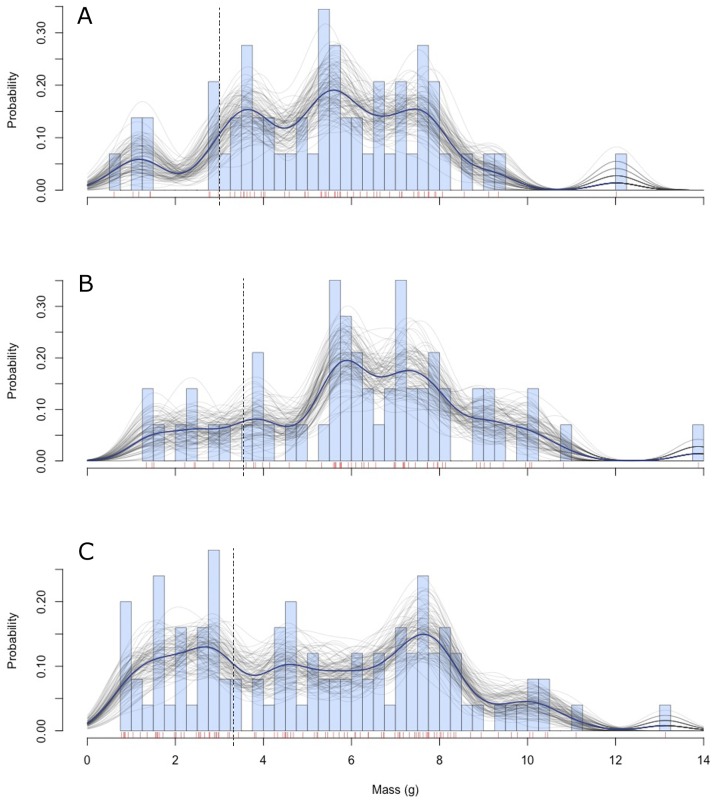
Frequency distributions of fruit sizes hoarded by A. *A*. *olivaceus*, B. *P*. *darwini* and C. *O*. *degus*. The dashed vertical lines indicate the mean size of fruits offered to each species during the trials (see text). Vertical marks along the seed mass axis represent unique documented hoarded fruits. We included a non-parametric smoothing spline fit (dark blue line) to the empirical distance distribution together with bootstrapped estimates (grey lines) to allow comparisons across plots.

**Fig 5:**
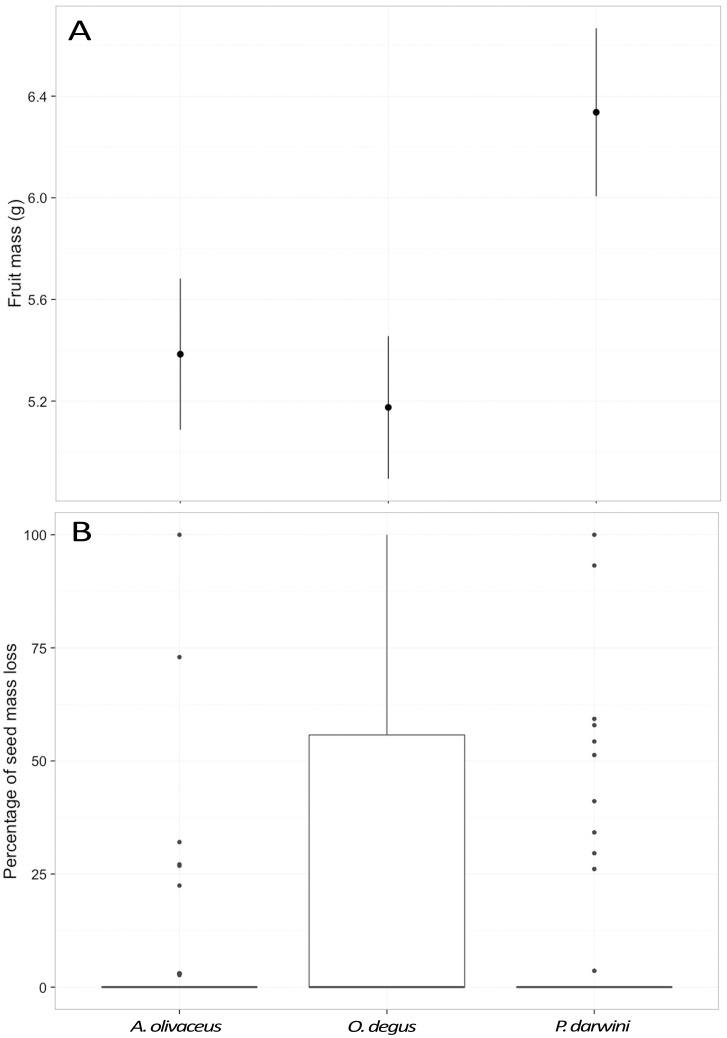
A. Fruit mass of fruits hoarded by *A*. *olivaceus*, *P*. *darwini* and *O*. *degus*. The graph shows means ± 1SE estimated using a mixed effects model. B. Boxplot of the percentage of seed mass consumed in predated seeds by *A*. *olivaceus*, *P*. *darwini and O*. *degus*.

We found that rodents of all three species were able to transport fruits within the entire range of sizes offered and that *O*. *degus* was the species that transported the most amounts of fruits (Fig 4). Mean size of hoarded fruits differed among rodents (*F*_*2*_ = 3.81, *P* = 0.02). We expected, on the basis of rodent body sizes, that the mean size of hoarded fruits would decrease with decreasing body size; however, we found that *P*. *darwini* hoarded the largest fruits, whereas *O*. *degus* and *A*. *olivaceus* hoarded fruits of approximately the same sizes (Fig 5A). Moreover, we found no effect of the fruit size/rodent size ratio on the percentage of fruits removed by *A*. *olivaceus* and *P*. *darwini* (*F*_1,17_ = 0.103, *P* = 0.75); this suggests, there were no upper limits to the seed sizes preferred by these two species. This relationship did not differ between rodents (*F*_1,17_ = 1.57, *P* = 0.23).

### Does fruit and seed size influence the probability of seed predation?

During the trials, rodents always consumed all of the pulp of the selected fruits and generally discarded intact seeds or only partially consumed them; they never discarded the pulp to consume only the seeds. Specifically, *A*. *olivaceus*, *P*. *darwini* and *O*. *degus* consumed only the fleshy pulp and discarded intact seeds of 84%, 82% and 59%, respectively of all the fruits they selected for consumption, the remaining percentages represent seeds that sustained some level of predation. The probability of a seed being predated differed among rodent species (χ^2^ = 27.88, d.f. = 2, *P* < 0.001), but did not vary in relation to fruit size (χ^2^ = 0.01, d.f. = 1, *P* = 0.92). Similarly, the percentage of seed mass lost did not vary as a function of seed mass (χ^2^ = 1.26, d.f. = 1, *P* = 0.26), but differed among rodents (χ^2^ = 17.54, d.f. = 2, *P* < 0.001) with *O*. *degus* consuming higher percentages of seed mass than either *A*. *olivaceus* or *P*. *darwini* (Fig 5B).

## Discussion

In this study we found that the size of *M*. *coquimbensis* fruits influences the foraging behavior of the scatter-hoarding rodents that consume its fruits and seeds. All three species of rodents harvested and hoarded the largest fruits (i.e., with higher mass); hence, they also dispersed the largest seeds. Patterns of fruit size preference were not affected by rodent size; even the smallest rodent species could transport fruits accounting for 50% of the body size. Finally, we found that fruit and seed size, however, had no effect on the probability of predation; contrary to what we predicted, larger fruits and seeds did not have lower predation probabilities.

Size is one of the most important fruit and seed traits, and its relationship with rodent foraging behavior has been extensively studied [1,4,11,12,17,18] because the decision to manipulate a fruit and/or seed is one of the most important from both the rodent and plant perspectives; moreover, its effects cascade down the successive steps in the foraging and scatter-hoarding process, and ultimately can influence seed dispersal dynamics [13]. Here, we found that as fruit size increased, so did the probability of it being harvested and hoarded. Similar results have been reported for fleshy-fruited species in the Atlantic forest of Brazil [18,19], for *Carapa procera* in French Guiana [10] and for rodent-dispersed Fagaceae in Southwest China [17] Rodent preferences for large fruits or seeds have been interpreted in terms of the optimal foraging theory because larger fruits and seeds offer higher nutritional rewards per foraging bout [20,21,50], thus they are more appealing to be consumed or stored as food [3,4,15] and water reserves [29]. Furthermore, given that *M*. *coquimbensis* occurs within an area of Atacama Desert where there are no other plant species that produce large fleshy fruits, it can be important for rodents to have large food reserves for the post-fruiting weeks, which is ultimately achieved by storing larger, rather than smaller fruits and seeds. Moreover, by hoarding larger fruits or seeds rodents make fewer caches, which is beneficial to the rodent for several reasons: 1) it is easier to remember where they are; 2) caches have a higher energetic content and; 3) fruits and seeds can remain stored for longer time periods [10,51,52]. Consequently, rodents may prefer storing seeds with higher nutrient content [53] in order to compensate for the energetic expenses incurred during the seed caching process (excavating, hiding, remembering, verifying, protecting and manipulating) [13]. Finally, it is worth noting that in addition to providing benefits to the rodents, hoarding the largest seeds may also contribute to increasing carbon storage in this area of the Atacama Desert because larger seeds produce larger plants [54], which have higher carbon storage potential [55]. Hence, scatter-hoarding rodents may be providing some of the ecosystem services typically associated with large-bodied frugivores, which are absent in this system [56].

In contrast to other studies that have reported that rodent body size may impose a limit on the size of fruits and seeds that can be transported [10,13,23,24], we found that for *A*. *olivaceus* and *P*. *darwini*, the fruit size/rodent size ratio did not have an effect on fruit choices. In a cafeteria experiment with 13 rodent and 42 plant species, respectively, Galetti and collaborators [19] proposed that rodent size limits fruit choices only when the fruit is the same size as the rodent. In our study, the highest fruit size/rodent size ratio was 0.5 (for *A*. *olivaceus*), and it did not appear to impose a limit on fruit transport. An unexpected result from this study was that the mean size of fruits hoarded by *O*. *degus* (the largest species) was not different from that of *A*. *olivaceus* (the smallest species), and smaller than that of *P*. *darwini*. This outcome is likely the result from the positive relationship observed between rodent size and the amount of fruits removed [29]; *O*. *degus*, being the largest rodent was also the species that removed the most amounts of fruits. Consequently, it hoarded a wider range of fruit sizes than *P*. *darwini*, which resulted in a lower mean size of hoarded fruits. Additionally, Jansen and collaborators [10] proposed that resource availability could be a factor that limits the preferential selection of large fruits because, when fruits are scarce, rodents will hoard everything that is available in order to survive periods of scarcity. Thus, it is possible that *O*. *degus* perceived low fruit abundance in the arenas (given its size) and hoarded most of what was available.

During our study, rodents typically ate only the fleshy pulp of the fruits offered and discarded whole, intact seeds, which suggests that the pulp may be satiating rodents before they consume the seeds. This result would be expected by the “quick meal” hypothesis [25,27], which states that the presence of fleshy pulp in rodent dispersed species is a trait that enhances caching by temporarily satiating the scatter hoarder and reducing seed predation. In our study, however, we did not find that the probability of a seed being predated decreased as fruit size increased; hence, it is unlikely that satiation explains why seeds were frequently discarded. One explanation for why rodents may initially favor the consumption of the pulp is that because they do not have access to drinking water in the area where they co-occur with *M*. *coquimbensis* (mean annual rainfall fluctuates around 80 mm), they must rely on preformed water in the diet, as well as in metabolic water to supply their needs [57]. Moreover, the fleshy pulp can provide additional nutrients [29,58] to those of the seed.

Although, in this study we did not explore whether pulp removal by rodents can alter the post-dispersal fate of seeds, there are at least two ways that it can affect the recruitment dynamics of *M*. *coquimbensis*. First, there is evidence that the pulp can inhibit germination if not removed [59], hence pulp removal would potentially increase the germination probabilities of dispersed seeds. Second, by removing the pulp rodents may alter the strength of subsequent interactions with other rodents [60], because when both fruits and seeds are available, rodents will prefer the fruits [26].

Within the subset of predated seeds, we did not find any evidence that larger seeds (i.e., with higher mass) satiated rodents more than smaller seeds; that is, the percentage of seed mass loss due to predation was not related to seed mass. However, the percentage of seed mass consumed differed among rodents; it was higher for *O*. *degus* than for the other two species. This suggests that the amount of seed mass needed to satiate a rodent is related to its body size and therefore, the larger the species, the more it would act as a seed predator instead of a disperser. Similar results have been reported by Galetti and collaborators [19], who found that the probability of seed predation increased with rodent body mass. These authors also classified predators into three functional groups based on their seed predation efficiency; using their framework, *P*. *darwini* and *A*. *olivaceus* would be considered as “inefficient seed predators” (from 5 to 20% of the seeds preyed upon; 18% and 16%, respectively) and *O*. *degus* as an “efficient seed predator” (41% of the seeds preyed upon). Nonetheless, of the seeds that were predated by *A*. *olivaceus*, *P*. *darwini* and *O*. *degus*, the former two consumed completely less than 2% of the seeds, and the last, less than 7%, revealing that these species frequently only partially consume the seeds. This is relevant for *M*. *coquimbensis* because this species can produce seedlings from seeds that have lost up to 90% of their mass, thus hoarded seeds that are retrieved and partially predated can still contribute the recruitment of this species [33,35].

Finally, it is important to consider that the relatively low seed predation rates observed in this study may be explained because the experimental trials only lasted four days, during which fresh fruits were available and hence rodents did not need to consume the seeds and thus acted more as seed dispersers. Nonetheless, it is possible that if only seeds were available—for example, at the end of the fruiting season or in years of low fruit production—rodents may act more as seed predators than dispersers [61]. Ultimately, the outcome of the interaction between *M*. *coquimbensis* and the three rodent species is likely context-dependent, and will be influenced by the relative abundance of each partner, the availability of alternate food sources, habitat structure and even soil moisture, which allows an easier detection of caches via olfaction [62,63].

In conclusion, our results reveal that larger *M*. *coquimbensis* fruits have higher probabilities of being harvested, and ultimately of its seeds being hoarded and dispersed by scatter-hoarding rodents. We found that the mean size of all hoarded fruits was 5.6 g, which is almost 60% larger than the mean fruit size along the species’ distribution range. Therefore, rodents may be acting as selective agents on *M*. *coquimbensis* fruit and seed size [16]; specifically, by consistently selecting the largest fruits rodents may either lead to the production of larger seeds over time or may at least contribute to the maintenance of large-seeded, fleshy fruits in an environment where producing them is costly. The selective role of seed predators on seed size is still controversial [64]; moreover, their selective effect may not translate into phenotypic selection if it is offset by factors operating later in the life cycle [65]. In *M*. *coquimbensis*, however, three lines of evidence point to the potential role of rodents as selective agents for this species. First, larger seeds not only have higher emergence probabilities, but also faster emergence times [54]. Second, seedling emergence and survival in the field is typically higher in the rock cavities where rodents hoard and cache the seeds [45]. Third, in spite of within individual variation in fruit and seed size, there are marked differences among plants in these traits [38], which suggests that they area heritable and may be shaped by the frugivore assemblage [66]. Ultimately, our results point to the key role of rodents in the regeneration of this endangered species and on understanding how fruit size can determine the outcome of this interaction.
